# An Individual with Both *MUTYH*-Associated Polyposis and Lynch Syndrome Identified by Multi-Gene Hereditary Cancer Panel Testing: A Case Report

**DOI:** 10.3389/fgene.2016.00036

**Published:** 2016-03-16

**Authors:** Stephanie A. Cohen, Christopher A. Tan, Ryan Bisson

**Affiliations:** ^1^Cancer Genetics Risk Assessment Program, St. Vincent HealthIndianapolis, IN, USA; ^2^Invitae CorporationSan Francisco, CA, USA; ^3^Cancer Genetics Center, UF Health Cancer Center–Orlando HealthOrlando, FL, USA

**Keywords:** next-generation sequencing, hereditary cancer, genetic counseling, *MUTYH*-associated polyposis, Lynch syndrome, case report

## Abstract

The utilization of next-generation sequencing technology to interrogate multiple genes simultaneously is being utilized more frequently in hereditary cancer testing. While this has benefits of reducing cost and allowing clinicians to cast a wide net in the elucidation of their patient's cancer, panel testing has the potential to reveal unexpected information. We report on a proband with pathogenic variants resulting in two different hereditary colon cancer syndromes. A 39-year-old male with a history of colon cancer, more than 20 colon polyps and a family history of colon cancer presented for genetic counseling. Testing with a 7-gene high-risk hereditary colon cancer panel identified a homozygous pathogenic variant, c.1187G>A (p.Gly396Asp) in *MUTYH*, and a likely pathogenic duplication of exon 7 in *MSH2*. Since this test result, the proband's mother was diagnosed with colon cancer; subsequent genetic testing confirmed she also carries the likely pathogenic duplication in the *MSH2* gene. Although the cancer risk in individuals who carry multiple pathogenic variants has not been established for combined biallelic *MUTYH*-associated polyposis and Lynch syndrome, the identification of multiple pathogenic variants does allow for screening for cancers associated with both syndromes and has implications for cancer risk for family members. In particular, this has significant impact on those who test negative for a known familial pathogenic variant, yet could be still be at risk for cancer due to a second pathogenic variant in a family. More information is needed on the frequency of occurrence of multiple pathogenic variants, as well as the phenotypic spectrum when multiple pathogenic variants are present.

## Introduction

A 39-year-old male who works as a general manager presented to the Cancer Genetics Risk Assessment program for genetic counseling due to a personal history of rectal cancer at the age of 38. In addition to the rectal cancer, more than 20 adenomatous polyps were found in his colon. He underwent a laparoscopic total proctocolectomy with ileoanal J-pouch anastomosis and diverting loop ileostomy. The tumor was a superficially invasive, moderately differentiated adenocarcinoma, arising within a background of adenoma and multifocal high-grade dysplasia. Immunohistochemistry staining as part of the hospital's universal screening program for Lynch syndrome displayed intact MLH1, MSH2, MSH6, and PMS2 proteins; microsatellite instability (MSI) testing was not performed. The proband's father had a history of basal cell carcinoma at the age of 58 and five colon polyps at the age of 61. Colon cancer was reported in the proband's paternal uncle at the age of 70 and a paternal great-grandmother in her 50s. Finally, the proband reported that his mother had a hysterectomy at a young age due to an unspecified gynecologic cancer.

**Figure 1:**
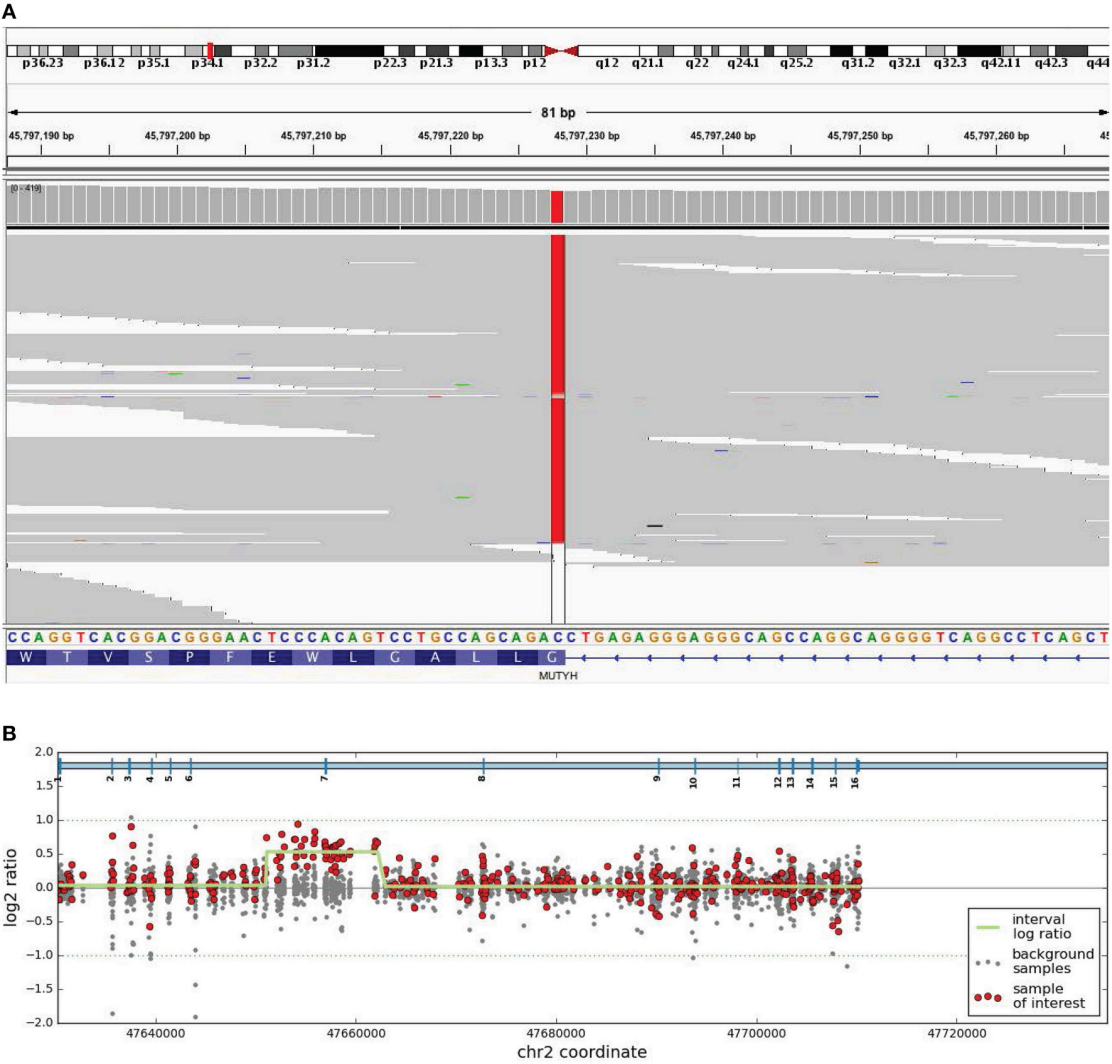
**Biallelic *MUTYH* mutation and likely pathogenic deletion in *MSH2*. (A)** Integrative Genomics Viewer images of next generation sequencing data of homozygous c.1187G>A (p.Gly396Asp) sequence variant with reference *MUTYH* nucleotide and amino acid nomenclature indicated. **(B)** Array CGH image. Red dots indicate the relevant clinical sample's log2 ratio confirming duplication of exon 7 in *MSH2*. Gray dots indicate the log2 ratios for seven other control samples. The green line indicates the segmentation by the DNA copy R package.

The proband underwent genetic testing with a 7-gene high-risk hereditary colon cancer panel which screened for variants in the *APC, EPCAM, MLH1, MSH2, MSH6, MUTYH*, and *PMS2* genes. The proband was found to carry a homozygous pathogenic variant, c.1187G>A (p.Gly396Asp) in *MUTYH*, and a likely pathogenic duplication of exon 7 in *MSH2* (Figure 1). The c.1187G>A (p.Gly396Asp) variant is a common cause of *MUTYH-*associated polyposis in individuals of Northern European ancestry and experimental studies have shown that this missense variant disrupts MUTYH protein function (Aretz et al., 2006; Ali et al., 2008). While the exact position of the exon 7 duplication in *MSH2* was not conclusively determined, the most likely explanation is that it occurs in tandem, which would likely result in a frameshift leading to a premature translational stop signal and an absent or disrupted MSH2 protein. A similar duplication of exon 7 in the *MSH2* gene has been reported previously in a patient affected with colorectal cancer (Casey et al., 2005). Both *MUTYH* and *MSH2* variants were confirmed by using an appropriate orthogonal method.

**Figure 2:**
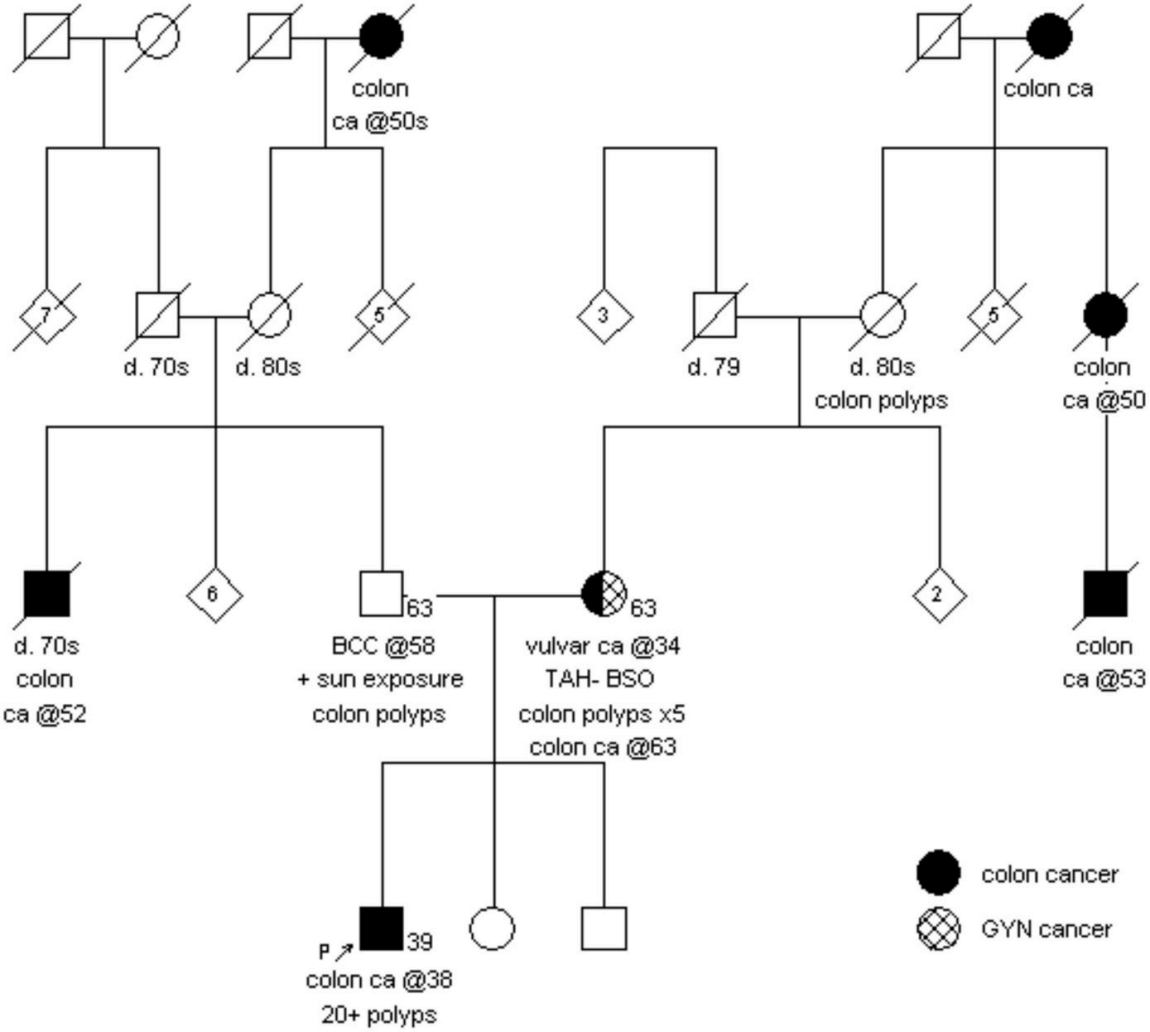
**Family history as reported by proband and his mother**.

The proband's parents subsequently underwent genetic counseling. The proband's mother clarified that she had a diagnosis of vulvar cancer at the age of 34 and five colon polyps at the age of 50. She reported a family history of colorectal cancer in her maternal grandmother at an unknown age, her maternal aunt at the age of 50 and a maternal first cousin at the age of 53; all of this was unknown at the time of the proband's initial genetic counseling appointment (Figure 2). While the proband's mother was waiting for her genetic test results, she was diagnosed with an invasive, well-differentiated mucinous adenocarcinoma of the cecum at the age of 63. The *MSH2* and *MSH6* proteins were absent by immunohistochemistry staining on the tumor, while *MLH1* and *PMS2* were preserved; MSI was not performed.

Genetic testing confirmed that each parent was heterozygous for the pathogenic variant c.1187G>A (p.Gly396Asp) in the *MUTYH* gene. The proband's mother was also found to carry the likely pathogenic duplication in the *MSH2* gene.

## Background

Lynch syndrome is an autosomal dominant condition caused by a pathogenic variant in one of four mismatch repair (MMR) genes or in *EPCAM*, a gene involved in epithelial cell adhesion. Lynch syndrome is characterized by an increased risk for colorectal cancer, as well as cancers of the endometrium, ovary, stomach, small intestine, hepatobiliary tract, urinary tract, and brain (Kohlmann and Gruber, 2004/2014; Cohen and Leininger, 2014). The lifetime risk for cancer among individuals with Lynch syndrome ranges widely, depending on the specific mismatch repair gene that is involved. The lifetime risk for colorectal cancer associated with a pathogenic variant in *MSH2* approaches 60%, and females with a pathogenic variant in *MSH2* have up to a 40% lifetime risk for endometrial cancer.

*MUTYH*-associated polyposis (MAP), an autosomal recessive condition caused by biallelic pathogenic variants in *MUTYH*, is characterized by multiple colorectal adenomas arising in adulthood and increased colorectal cancer risk. Lifetime risk for colorectal cancer among patients with biallelic pathogenic variants in *MUTYH* is estimated to be at least 43 to almost 100% in the absence of timely surveillance (Maartje Nielsen et al., 2012/2015; Lubbe et al., 2009). Biallelic pathogenic variants in *MUTYH* have also been described in Lynch and Lynch-like families in the absence of pathogenic variants in the MMR genes (Castillejo et al., 2014). Whether monoallelic pathogenic variants in *MUTYH* also increase cancer risk is unclear, although there is some evidence suggesting a slight increase, especially when there is a family history of colon cancer (Win et al., 2014). The risk for colorectal cancers for carriers of a pathogenic variant in one of the MMR genes in addition to a single *MUTYH* pathogenic variant was substantially higher than that for carriers of a single *MUTYH* pathogenic variant alone, but not different from that for carriers of a MMR pathogenic variant alone (Win et al., 2015).

Cases of multiple hereditary cancer syndromes in a family caused by pathogenic variants in different genes related to two hereditary cancer syndromes have been previously reported in the literature (Thiffault et al., 2004; Gong et al., 2012). The cumulative cancer risks for patients who have both MAP and Lynch syndrome are not well characterized. There is one previous report of a patient identified with compound heterozygous pathogenic variants in *MUTYH* who also carried a pathogenic variant in *MSH6;* this individual exhibited a relatively mild clinical presentation (van Puijenbroek et al., 2007). To our knowledge, biallelic *MUTYH* pathogenic variants in addition to a pathogenic variant in *MSH2*, as described in our patient, have not been previously reported.

## Discussion

This case demonstrates several points about genetic testing in the era of next generation sequencing (NGS) panel testing. First, had an immunohistochemistry-guided approach to testing been followed for the proband, the likely pathogenic variant in *MSH2* would have been missed. It appears in this case that the proband's rectal cancer was a result of the biallelic pathogenic variants in the *MUTYH* gene, which would not have resulted in absent *MSH2* staining in his tumor. Conversely, his mother's tumor appears to be a result of a MMR pathway defect that did result in absent *MSH2/MSH6* staining. A panel was chosen for the proband in this case due to his diagnosis of colon cancer at a very young age, the presence of colon polyps, the presence of colorectal cancer in his father's family and the possible gynecologic (at the time unknown) cancer in his mother. There is some recent evidence that the incidence of pathogenic variants among individuals diagnosed with colon cancer at a very young age is high, with as many as 14–35% of individuals with colon cancer under age 35 found to carry a pathogenic variant in a variety of different genes (Chubb et al., 2015; Mork et al., 2015). Even considering a broader age range with diagnoses of colon cancer up to the age of 50, as many as 15% had an underlying pathogenic variant in a hereditary cancer gene (Pearlman et al., submitted).

The inheritance patterns for these two genetic conditions are different. Lynch syndrome is autosomal dominant and MAP is autosomal recessive. This has significant implications to family members. Had only the biallelic pathogenic variants in the *MUTYH* gene been identified, the proband's children would have been falsely reassured that they were just carriers of a single *MUTYH* variant. Surveillance recommendations for individuals who are carriers of a single *MUTYH* pathogenic variant are not well defined, but typically would include colonoscopy every 5 years, similar to recommendations for individuals with a first-degree relative with colorectal cancer (Stoffel et al., 2014; Provenzale et al., 2015). Surveillance for individuals with a pathogenic variant in *MSH2* is very different, with annual colonoscopy recommended beginning at age 25 (Provenzale et al., 2015; Syngal et al., 2015). In addition, surveillance for other cancers may be considered, such as upper endoscopy, due to the risks for other cancers associated with Lynch syndrome. Finally, females are at increased risk for endometrial and ovarian cancers with Lynch syndrome, so hysterectomy after childbearing should be considered. As there is variable expression of MAP and Lynch syndrome in general, the identification of additional patients with combined diagnoses would be necessary to make more definitive conclusions regarding cumulative cancer risks.

The proband's extended family is also impacted by this complex test result. The determination of the *MSH2* duplication as maternally inherited has implications for maternal family members, while the cause of the colon cancer in paternal family members remains unexplained, which may prompt family members to undergo further analysis. Communication among family members is very important, especially in cases where multiple pathogenic variants are identified. There are at least three affected family members for every person identified with Lynch syndrome (Hampel et al., 2008). If this is true when there are multiple pathogenic variants present, then many more relatives could potentially be impacted. Sharing of test results among family members and their doctors is critical to identification of other family members at risk and recommendations for appropriate surveillance.

As more genetic testing is completed with NGS panels, we anticipate a rise in the identification of cases of multiple pathogenic variants. It is also important for clinicians to be mindful of the possibility of a second pathogenic variant in a family where one pathogenic variant has already been described, particularly in cases where the inheritance pattern may not match the known pathogenic variant (for example, if the inheritance appears to be autosomal dominant, but a recessive condition like MAP is documented), or if the clinical picture doesn't match the known pathogenic variant.

## Concluding remarks

Although the cumulative cancer risk in individuals with a combined diagnosis of MAP and Lynch syndrome is unknown, the identification of multiple pathogenic variants does allow for screening for cancers associated with both genes and has implications for cancer risk and surveillance recommendations for family members. In particular, this has significant impact on those who test negative for a known familial pathogenic variant, yet could be still be at risk for cancer due to a second pathogenic variant in a family. More information is needed to understand the frequency of occurrence of multiple pathogenic variants, as well as the phenotypic spectrum when more than one syndrome is present.

## Author contributions

SC, CT, and RB contributed to the acquisition of the information for this case report. SC drafted the manuscript, and it was reviewed and approved by all three authors.

## Ethics

Informed written consent was obtained from the proband, his mother and father for genetic testing and from the proband and his mother for inclusion in this case report.

### Conflict of interest statement

CT is an employee of Invitae Corporation, a testing laboratory that furnished the 7-gene panel test results used in this study. The other authors declare that the research was conducted in the absence of any commercial or financial relationships that could be construed as a potential conflict of interest.
